# Tracing the Uncharted African Diaspora in Southern Brazil: The Genetic Legacies of Resistance in Two *Quilombos* from Paraná

**DOI:** 10.3390/genes16121510

**Published:** 2025-12-16

**Authors:** Iriel A. Joerin-Luque, Isadora Baldon Blaczyk, Priscila Ianzen dos Santos, Ana Cecília Guimarães Alves, Natalie Mary Sukow, Ana Carolina Malanczyn de Oliveira, Thomas Farias de Cristo, Angela Rodrigues do Amaral Bispo, Aymee Fernanda Gros, Maria Letícia Santos Saatkamp, Victor Dobis Barros, Joana Gehlen Tessaro, Maria Eduarda da Silveira Costa, Luana Leonardo Garcia, Isabela Dall Oglio Bucco, Denise Raquel de Moura Bones, Sarah Elisabeth Cupertino, Letícia Boslooper Gonçalves, Alaerte Leandro Martins, Gilberto da Silva Guizelin, Adriana Inês de Paula, Claudemira Vieira Gusmão Lopes, Marcia Holsbach Beltrame

**Affiliations:** 1 Programa de Pós-Graduação em Genética, Departamento de Genética, Universidade Federal do Paraná (UFPR), Curitiba 81531-990, Paraná, Brazil; irieljoerin@gmail.com (I.A.J.-L.);; 2 Laboratório de Genética Molecular Humana, Departamento de Genética, Universidade Federal do Paraná (UFPR), Curitiba 81531-990, Paraná, Brazilmarialsaatkamp@gmail.com (M.L.S.S.); sarah.esc.95@gmail.com (S.E.C.); 3 Programa de Pós-Graduação em Medicina Interna, Hospital de Clínicas (HC), Universidade Federal do Paraná (UFPR), Curitiba 81531-990, Paraná, Brazil; 4 Laboratório de Imunogenética e Histocompatibilidade, Departamento de Genética, Universidade Federal do Paraná (UFPR), Curitiba 81531-990, Paraná, Brazil; 5 Rede Mulheres Negras do Paraná (RMN-PR), Curitiba 80310-130, Paraná, Brazil; 6 Programa de Pós-graduação em História, Universidade Federal do Paraná (UFPR), Curitiba 81531-990, Paraná, Brazil; 7 Departamento de Educação Física, Universidade Federal do Paraná (UFPR), Curitiba 81531-990, Paraná, Brazil; adrianaines@ufpr.br; 8 Setor Litoral, Universidade Federal do Paraná (UFPR), Matinhos 83260-000, Paraná, Brazil

**Keywords:** human migration, uniparental markers, gene flow, phylogeography

## Abstract

Background/Objectives: In Brazil, *quilombos*—African-descendant resistance communities—emerged during slavery and persisted beyond its abolition. The state of Paraná, in Southern Brazil, is home to 86 *quilombos*, yet their genetic diversity remains entirely unexplored, and little is known about their subcontinental African origins. Methods: To explore the demographic history of these communities and the reach of the Transatlantic Slave Trade in Southern Brazil, we analyzed Y and mitochondrial DNA haplotypes in samples from two *quilombo* communities from Paraná, Feixo (*n* = 117) and Restinga (*n* = 47). Results: Our findings reveal a significant African maternal ancestry in both communities, with Feixo exhibiting 35% and Restinga showing a striking 78.72% of maternal haplogroups of African origin. Feixo’s mtDNA haplotypes display affinities with Bantu-speaking populations from Central-Western and Southeastern Africa (such as Angola, Congo, and Mozambique), whereas those found in Restinga are more closely aligned with lineages frequent in Western Africa. Y-chromosome data reveal 39.4% and 25% African paternal ancestry in Feixo and Restinga, respectively, with most African chromosomes assigned to haplogroup E1b1b1-M35, which has a broad frequency across eastern Africa. Conclusions: These results offer novel insights into the history of the African diaspora in a previously unstudied Brazilian region, suggesting African sources—including underdocumented Eastern/Southern lineages—and contributing useful new clues to their broader within-Africa affinities.

## 1. Introduction

The transatlantic traffic of human beings triggered an unprecedented diaspora in the history of humanity. It has been estimated that more than 12 million Africans were forcibly relocated to America, Europe, and the Atlantic islands [1]. Throughout the continent, Africans and their descendants developed systems of resistance, some of which gave rise to communities such as *quilombos* in Brazil and *cumbes*, *palenques*, or *maroons* elsewhere in Latin America [2]. Despite receiving nearly half of the Africans brought to America, Brazil still presents the lowest transatlantic traffic data coverage [3].

In Brazil, Africans and their descendants established *quilombos* during slavery and shortly after its abolition in 1888 [2]. Currently, there are 7666 *quilombos* distributed across almost the entire country, encompassing over 1.3 million *quilombola* individuals, according to the latest 2022 Brazilian Census [4]. In this context, *quilombos* developed as a form of resistance to the oppression of slavery in colonial society [5]. Their formation often involved other ethnic groups, such as neighboring Indigenous American populations and other marginalized peoples [5,6]. Some of these communities settled in isolated or rugged areas to preserve their autonomy and freedom [2]. However, as each *quilombo* developed within distinct environmental and historical contexts—and was founded by people originating from diverse ethnolinguistic backgrounds—their individual trajectories collectively contribute to reconstructing the broader narrative of the largest diaspora in human history [5,7].

Based on a longstanding eugenic ideology, the white oligarchy of the state of Paraná constructed an official history that hid the involvement of the African workforce during the region’s development [8]. It was not until recent years (2005–2010) that the Clóvis Moura Working Group (Grupo de Trabalho Clóvis Moura), a governmental organization, made visible the 86 African-derived rural communities that are distributed across the territory, particularly in regions that historically benefited the most from slavery (Grupo de Trabalho Clóvis Moura, 2010). In line with these historical accounts, the most recent Brazilian demographic census (2022) demonstrated that the cities of Palmas and Lapa comprise the most significant proportion of the *quilombola* population, which constitutes 0.06% of the total inhabitants of the state (representing 7113 people) [4]. According to the same source, there are currently 38 officially certified *quilombos* in Paraná.

**Figure 1:**
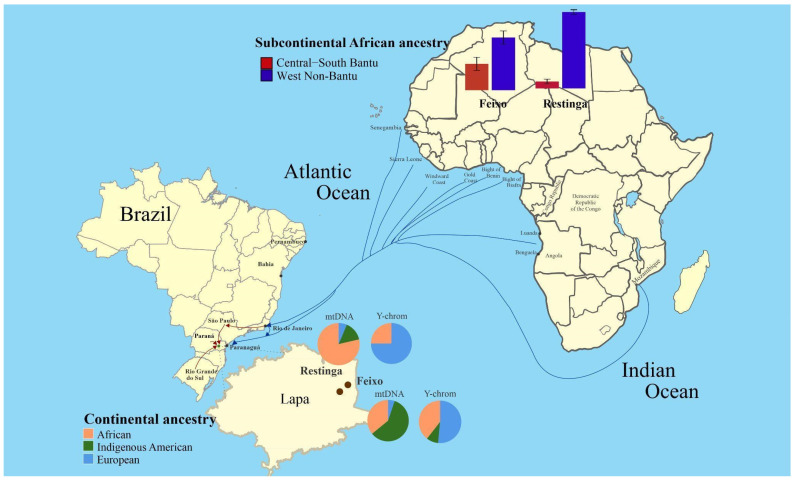
Geographical location of the African-derived populations Feixo and Restinga and uniparental ancestry proportions observed. The region of Lapa, in the state of Paraná -where the *quilombos* Feixo and Restinga are located- is enlarged on the map. Uniparental ancestry proportions observed in each community are shown in pie charts. Blue arrows indicate possible slave vessel routes from the main coast of departure during the transatlantic traffic and to the *quilombos*, based on historical records [43,44]. Red arrows outline possible land routes for the entry of Africans enslaved to the *quilombos* based on historical records [8,13]. Bar charts show the estimated proportions of sub-continental African maternal ancestry calculated according to the number of haplotypes from each African region for both *quilombos*.

Feixo and Restinga are two of these *quilombos*, located in the Lapa region (Figure 1). They were founded shortly before the abolition of slavery in 1888 by Black families who had been enslaved on two plantations in the area [9]. Their founders received portions of the lands where they had worked, upon which the *quilombos* were established [9].

Unlike other more isolated or semi-isolated *quilombos* in Brazil, Feixo and Restinga have maintained intense interactions—particularly in more recent times—with neighboring populations, especially with the nearby Mariental colony, but also with the city of Lapa and the capital of the state, Curitiba [9,10]. They also preserve oral histories recounting that some residents migrated from one *quilombo* to the other.

In Brazil, African ancestry ranges from 10.9 to 28.8%, on average, in urban regions; however, it is worth noting that samples from these urban areas might be somewhat biased due to a low number of Black participants, and 40–90%, on average, in the *quilombos* studied so far (very few of which are from the southern region of Brazil) [7,11]. However, most of the population has yet to discover the specific origin of their African ancestors [1]. The settlement of the Lapa region was closely related to *tropeirismo*—a form of stockbreeding and cattle transport between southern and southeastern Brazil—suggesting that several enslaved Africans, either from São Paulo or from Rio de Janeiro cities, may have arrived in the region through these routes [12]. It is believed that most Africans who arrived in São Paulo came through the port of Rio de Janeiro, which primarily received enslaved people from Angola, Congo, and, after 1845, from Mozambique [12]. However, after 1850, São Paulo also received large contingents of West Africans relocated through the interstate traffic of enslaved people between northeastern and southeastern Brazil, which continued until the abolition of slavery [2]. Nonetheless, there are no specific records regarding the African ethnolinguistic origins of the enslaved population in Lapa [13].

Population differences at the DNA level have long been investigated using a variety of genetic systems, including forensic short tandem repeats (STRs), which have generated extensive allele frequency datasets in human populations worldwide [14]. Previous genetic studies of Brazilian *quilombos* have relied mainly on uniparental markers (mtDNA and Y-chromosome) to explore sex-biased admixture patterns and the African, Indigenous American, and European contributions to these communities [15,16,17,18,19,20]. These analyses have generally shown high African ancestry in *quilombos* compared with surrounding urban populations, but also marked heterogeneity among regions and communities, as well as recurrent patterns of African and Indigenous American maternal lineages and European paternal lineages [15,16,19,20,21,22,23,24,25]. However, most of this work has focused on *quilombos* from the North and the Northeast of Brazil, with only a few communities from the South and Southeast—and none from the state of Paraná—characterized so far, leaving an important gap in our understanding of how the African diaspora unfolded in this part of the country. For a comprehensive overview of genetic studies in Brazilian *quilombos*, see Joerin-Luque et al., 2023 [7].

Here, we leveraged the genetic data obtained from uniparental markers (mitochondrial DNA and Y chromosome SNPs), together with historical records and published African haplotype data, to explore, for the first time, the phylogenetic links between the *quilombos* Feixo and Restinga and Africa. Through this work, we contribute to the knowledge of the origins and trajectories of the African diaspora in Southern Brazil, highlighting differences in African contributions to Feixo and Restinga that are compatible with differentiated historical and demographic processes for these communities.

## 2. Materials and Methods

### 2.1. Sample Collection and DNA Extraction

This study was approved by the local institutional ethical review board, Comitê de Ética em Pesquisa em seres humanos, Setor de Ciências da Saúde, CEP/SD (CAAE number 42761521.9.0000.0102). Written informed consent was obtained from all participants for sample collection and interviews. Blood samples were collected along with ethnographic information (name, sex, age, place of birth, and self-declared color). Self-declared color data were collected using the Brazilian Institute of Geography and Statistics (IBGE) categories: Preto, Pardo, Branco, Amarelo, and Indigena, translated here as Black, Admixed, White, Asian, and Indigenous [4]. In alignment with the demands of the Brazilian Black Movement, the categories Preto and Pardo are often aggregated under the broader term Negros (Black individuals) to reflect shared experiences of racial inequality [26]. We also collected data on family history (self-declared color and parents’ place of birth). DNA was extracted from the buffy coat of blood samples using a commercial kit following the manufacturer’s instructions (Wizard Genomic DNA Purification Kit, Promega Madison, WI, USA). All analyses were performed anonymously.

### 2.2. Genotyping

Overall, 164 samples (Feixo *n* = 117, Restinga *n* = 47) were genotyped for 6 mtDNA-coding-region single-nucleotide polymorphisms (SNPs)—827, 1736, 5178, 10873, 10400, and 15487—through allele-specific PCR and the product size was confirmed by agarose gel electrophoresis [27]. After the initial screening, samples with the ancestral state for positions 10873 and 10400, which identify clade L, were selected for sequencing of the control region of the mitochondrial genome—D-loop—(see Sanger sequencing section). Male samples (*n* = 45, Feixo *n* = 33, Restinga *n* = 12) were genotyped for 5 Y chromosome SNPs—M242, M207, M89, M9, and P152- through allele-specific PCR with the primers described by Medina and colleagues [28]. PCR product sample sizes were confirmed by agarose gel electrophoresis. Samples positive for the derived state of M242 were also tested for M3 and M346 mutations to check the sub-lineage of Native American haplogroup Q [28]. Samples positive for the derived state of P152 were additionally tested for SNPs M35 and M2. PCR primers for allele-specific PCR are described in Table S1.

### 2.3. Sanger Sequencing

MtDNA control hypervariable regions 1 to 3 (HVRI, HVRII, and HVRIII) were sequenced using Sanger’s Method. HVRI was PCR-amplified with primers L15997 (5′-CACCATTAGCACCCAAAGCT-3′) and H017 (5′-CCCGTGAGTGGTTAATAGGGT-3′), whereas HVRII-III was amplified with primers L034 (5′ CCATGCATTTGGTATTTTCG-3′) and H629 (5′-TTTGTTTATGGGGTGATGTGA-3′) [29]. The PCR products were purified with the enzymes exonuclease I (Fermentas, Vilnius, Lithuania) and alkaline phosphatase (Thermo Fisher Scientific, Waltham, MA, USA) on a Mastercycler EP Gradient S (Eppendorf) at 37 °C for 1 h and 80 °C for 15 min. The purified products were then amplified using BigDye Terminator Cycle Sequencing Standard v3.1 (Life Technologies, Carlsbad, CA, USA), according to the manufacturer’s instructions, and using the same primers. The sequencing thermocycler program consisted of a first step at 95 °C for 1 min, followed by 25 cycles of 95 °C for 10 s, 50 °C for 5 s, and 60 °C for 4 min. The products were purified using ethanol (Merck, Darmstadt, Germany), resuspended in Hi-Di Formamide (Life Technologies), and, finally, submitted to capillary electrophoresis in a 3500xl Genetic Analyzer Sequencer (Life Technologies).

### 2.4. Bioinformatic Analysis

MtDNA control region electropherograms were aligned to the rCRS (NC_012920.1) [30,31]. Polymorphic positions were analyzed using Mutation Surveyor R^©^ 3.30 (SoftGenetics, State College, PA, USA). MtDNA fasta sequences retrieved from GenBank for comparisons were aligned to the rCRS using the software Mega v11.0.13 [32]. Haplogroup assignment was done using EMPOP (database version v4/R13) and HaploGrep3 [33]. Haplogroup frequencies were calculated by direct counting. Ethnographic information available—kinship until first grade—was used to double-check the haplotypes observed. Positions with missing data and InDels (especially from poly-C regions from positions 303–315, 523–524, and 16183–16194) were removed from all analyses. For the construction of haplotype networks, we retrieved from the literature the GenBank codes of mitochondrial sequences matching two criteria: 1—Be assigned to one of the haplogroups detected in our sample; 2—Have been collected in populations from the African regions known to be involved with the slave trade to Brazil. Despite the absence of records of enslaved Africans brought to Brazil from Madagascar, sequences from that location were also used in the comparison as proxies to the Bantu lineages found in Mozambique, known to be a contributor to Madagascar and Brazilian gene pools [34,35,36]. In the same line, samples from Morocco were used as proxies for lineages from unsampled populations from West Africa. Haplotype parsimony networks and indices of haplotype (*H*) and nucleotide diversity (π) were calculated with the R package *pegas* v1.3 [37]. Analysis of molecular variance (AMOVA) was performed using the R package *poppr* v2.9.5 [38]. Correspondence analysis was performed using the function CA of the R package *FactoMineR* v2.9 and visualized with the package *factoextra* 1.0.7 [39]. Because publicly available mtDNA data from Africa are unevenly distributed, the reference populations included in the correspondence analysis were used as regional proxies. These groups correspond to the African populations historically documented as contributors to the Transatlantic Slave Trade with Brazil.

## 3. Results

The mtDNA and Y chromosome haplotypes detected in Feixo and Restinga are described in Tables S2 and S3, along with the corresponding haplogroup classifications. The EMPOP algorithm and HaploGrep v.3 classified the haplotypes identically (Table S2).

### 3.1. The Maternal Component: Continental Insights into Genetic Ancestry

We genotyped 164 samples (Feixo *n* = 117, Restinga *n* = 47) for 6 mtDNA SNPs (10400, 10873, 1736, 827, 15487, and 5178), which define the macro-haplogroups M and N, and the clades A, B4b’d’e’j, M8 (ancestral to clade C), and D, respectively. Clades A, B4b’d’e’j, M8, and D are ancestral to all Native American founder Hgs (A2, B2, C1b, C1d, C1c, C4c, D1, D2a, D3, D4h3a, D4e1c, X2a, and X2g) [40,41]. We considered all mitochondrial chromosomes positive to A, B4b’d’e’j, M8, and D markers to be Native American (from A, B, C, and D clades, respectively) as the intermediate Hgs between those ancestral clades, and the Indigenous American ones only occur in Asian populations, which are not reported to be involved in the formation of *quilombos* [2]. For the same reason, as all the mitochondrial lineage diversity from out of Africa derives from macro-haplogroups M and N, haplotypes attributed to them (but not to A, B, C, and D clades) were considered of European origin [42]. MtDNA chromosomes with the ancestral states for 10873 (N) and 10400 (M) were attributed to the African macro-haplogroup L.

Considering both communities, most haplogroups were African or Indigenous American (African = 46.9%, Indigenous American = 47.5%). However, when we observed each community separately, a clear majority of African lineages was observed in Restinga (78.7%), whereas Feixo presented mostly Indigenous American Hgs (60.9%) with a 33.9% of African ancestry (Figure 1). The European contribution to the maternal lineages was less expressive in both *quilombos* (European = 5.6%) (Table 1).

### 3.2. Distinct Patterns of Indigenous American Haplogroup Distribution in Feixo and Restinga

Only Indigenous American clades A, B, and C were detected in Feixo and Restinga, and a closer look at their Hg composition highlights significant differences between them (Fisher exact test *p*-value = 0.02453). In Feixo, the striking majority of lineages belonged to Hg C (46.1% of the total Hgs), whereas in Restinga, the highest proportion corresponded to Hg A (8.5% of the total Hgs) (Table 1).

### 3.3. Feixo and Restinga May Have Received Distinct African Genetic Contributions

Overall, 76 samples were assigned to mitochondrial African clades (L-derived) according to the genotyping of SNPs 10400 and 10873. For all those samples, we sequenced the mitochondrial D-loop to establish the specific lineage to which they belong (Feixo *n* = 39, Restinga *n* = 37) (Table S2). To help elucidate the historical sources of the African contributors to the maternal lineage pool of Feixo and Restinga, we employed a phylogeographic approach to compare their haplotypes with those reported from African source populations in the published literature (Table S4).

**Figure 2:**
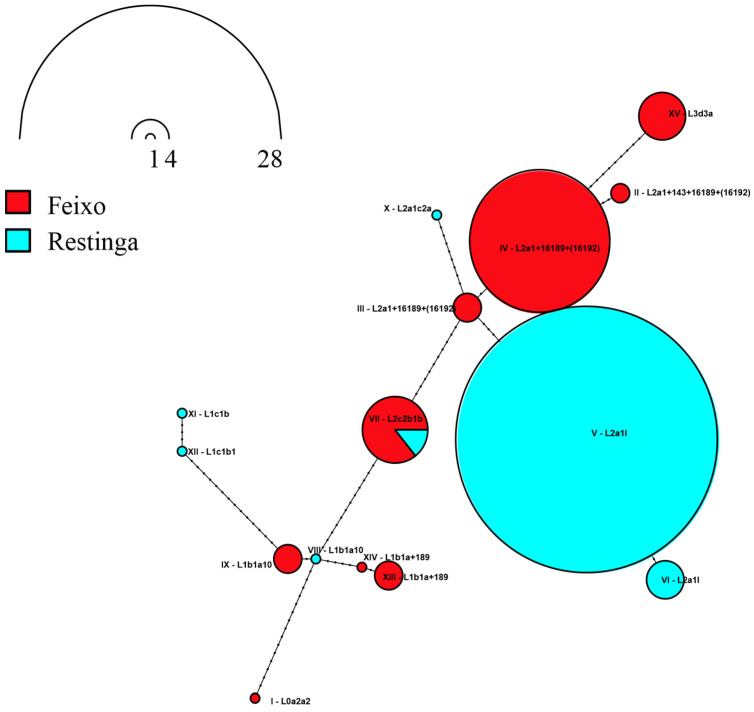
Haplotype network of African mitochondrial haplotypes detected in Feixo and Restinga. Haplotypes are identified with Roman numerals. Black circles on the network links represent mutational steps between the haplotypes.

Only sub-branches of clades L0–L3 were detected in both communities, comprising 12 haplogroups (Table 1). The predominant haplogroups were lineages derived from L2 (78.9%) and L1 (13.2%) (Table 1 and Figure 2). From clade L1, only lineages derived from L1b were detected in Feixo, whereas lineages derived from L1b and L1c were present in Restinga (Table 1 and Figure 2).

In Feixo, we detected two lineages derived from Hg L1b1a: L1b1a+189 and L1b1a10. When compared with the haplotypes belonging to the same haplogroup from African reference populations (Table S4), the sample L1b1a10 found in Feixo matched haplotypes detected in Bantu from Angola and in Niger-Congo non-Bantu samples from Nigeria, and Burkina Faso (belonging to Yoruba, Gur, and Mande branches) (Figure S1). Haplotypes from Hg L1b1a+189 did not match any haplotypes from the populations evaluated but were similar to one haplotype found in the Mandenka sample from Senegal (Figure S1).

The lineages L1c1b and L1c1b1 were detected exclusively in Restinga (Table 1 and Figure 2). Those lineages have been proposed as originating in Bantu or pre-Bantu populations [45]. Consistently, one of the haplotypes identified in our sample was identical to those found in Bantu samples from southwestern Angola and Zambia, as well as in Khoe-Kwadi speakers and hunter-gatherer groups from the same region, who have been shown to be genetically related to their Bantu neighbors (Figure S2) [46]. The haplotype L1c1b1 found in Restinga did not match any of the sequences evaluated. However, it was similar to an ancestral L1c1b from Zambian Bantus, compatible with a Southern Bantu origin for these lineages in Restinga (Figure S2).

Clade L2 was the most frequent overall (66.7 in Feixo and 91.9% in Restinga), but only two out of the five main L2-derived clades (L2a, -b, -c, -d, and -e) were detected in the *quilombos*. Specifically, lineages derived from haplogroups L2a1 and L2c2b1b were identified (Table 1 and Figure 2).

In Feixo, we detected the L2a1-derived lineages L2a1+143+16189+(16192) and L2a1+16189+(16192), the latter occurring at a high frequency (46.2%) (Table 1). None of the haplotypes identified in Feixo matched those reported in the African reference populations evaluated. However, they clustered closely with Moroccan and Burkina Faso non-Bantu individuals, consistent with a possible Western African non-Bantu origin (Figure S3).

The *quilombo* Restinga exhibited a very high frequency of Hg L2a1l, comprising the majority of identified haplotypes in that community (86.5%). Interestingly, all L2a1l chromosomes were represented by only two haplotypes that differ by the presence of the hotspot mutation 16519T (Figure 2) [47,48]. This finding is consistent with a single founding haplotype in Restinga (haplotype II in Figure S4, of higher frequency), in which the 16519T transition emerged (Figure 2). The comparison with L2a1l haplotypes from African populations is consistent with a source of this lineage in West/North Africa, as the haplotypes found in Restinga matched haplotypes from samples from Burkina Faso non-Bantu and Moroccan populations (Figure S4). Other minor haplogroups were L2a1c2a and L2c2b1b. The haplogroup L2a1c2a was found exclusively in Restinga. Although the observed haplotype did not match any previously published sequences, it was only one mutational step away from haplotypes reported in populations from Burkina Faso (Figure S5). The L2c2b1b haplotypes observed in Feixo and Restinga (Figure 2) only differ from those found in Angola, South Africa, Zambia, and Madagascar by the presence of the hotspot mutation 16519C (Figure S6), compatible with a Central/Southern African Bantu origin for that lineage.

L0a2a2 was the only L0 lineage detected in these *quilombos* at a low frequency (2.6%) and exclusively in Feixo (Table 1 and Figure 2).

Finally, the lineage L3d3a was also detected exclusively in Feixo, at 12.8% (Table 1 and Figure 2). Also, the haplotype network with Southern African samples showed a match of the haplotypes found in Feixo with the several available L3d3a sequences of Bantu and Khoe populations from Angola, Namibia, Zambia, South Africa, and Botswana (Figure S7).

### 3.4. Different Patterns of Sub-Continental Ancestry Are Observed in Both Quilombos

To further explore the general patterns of sub-continental African ancestry in each *quilombo*, we considered the frequency of the Hgs detected and compared them with that of African populations from regions known to have contributed to the African diaspora in America through a Correspondence Analysis (CA). Due to the high number of mtDNA Hgs described to date, we used a hierarchical approach to reduce this number (reference populations and their Hg frequencies are described in Table S5).

**Figure 3:**
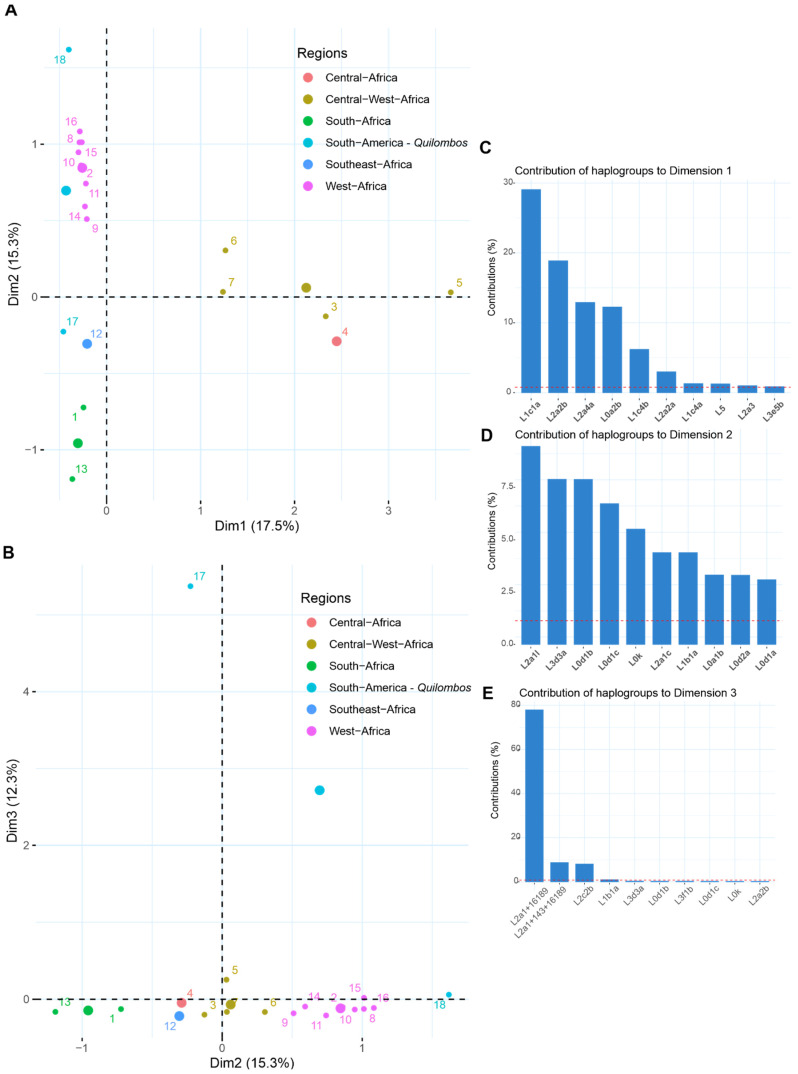
Correspondence Analysis of mtDNA haplogroup composition in Feixo, Restinga, and African reference populations. (**A**) Dimensions 1 and 2. (**B**) Dimensions 2 and 3. (**C**–**E**) Contribution of haplogroups to each dimension. 1: Angola, 2: Burkina Faso, 3: Cameroon, 4: Central African Republic, 5: Congo, 6: Equatorial Guinea, 7: Gabon, 8: Gambia, 9: Ghana, 10: Guinea-Bissau, 11: Ivory Coast, 12: Mozambique, 13: Namibia, 14: Nigeria, 15: Senegal, 16: Sierra Leone, 17: Feixo, 18: Restinga.

As expected, reference African populations clustered according to the geographical distribution of mitochondrial Hgs, from North to South along the second dimension, which captures 15.3% of the variance (Figure 3A,B). The first dimension accounted for 17.5% of the variance and mainly separated Central African populations in a gradient from west to east (Figure 3A,B). In this layout, Feixo and Restinga adopted very divergent positions. Whereas Feixo was closer to the Central/Southern/Southeastern populations, Restinga clustered closely with the Western African ones. Looking at the haplogroups with the highest contributions to the CA dimensions, it is possible to see that the arrangement of the populations along the second dimension is mainly influenced by the frequencies of Hgs L2a1l, which approximates Restinga to Western populations and by L3d3a, which approximates Feixo to Southern populations (Figure 3A,B,D and Figure S8). L0-derived lineages, enriched in Southern Africa, also contribute to the North/South separation of the populations and their clustering with Feixo (Figure 3A,B,D and Figure S8).

When a third dimension is included (Figure 3B,E), we observed that lineages L2a1+143+16189 and L2a1+16189 differentiate Feixo from the other populations. This divergence could be attributed to genetic drift, but we must also consider that the presence of homoplasic positions (143, 16189, 16192, and 16309) might complicate the accurate haplogroup assignment of these samples [49].

To further explore the demographic history of these communities, we compared the African maternal lineages of Feixo and Restinga with those of other *quilombos* and urban Brazilian populations (Figures S1 and S2). Notably, no clear geographical clustering emerged among *quilombos*, despite their distribution across Brazil. Instead, their mitochondrial haplogroup composition—limited to the most frequent Sub-Saharan African clades (L0a, L1, L2, and L3)—varied distinctly between communities. For example, Feixo and Restinga grouped more closely with the Northern *quilombo* of Marajó than with urban samples from Southern Brazil, which instead clustered with urban populations from the Central-Western and Southeastern regions (Figures S1 and S2).

### 3.5. The Male Lineages in Both Quilombos Were Mainly European

We genotyped 45 samples (Feixo *n* = 33, Restinga *n* = 12) for 9 Y SNPs. Most Y chromosomes were allocated to clade R-M207 (42.2%). Clade R-M207 is the most common in the urban admixed populations of Brazil and predominant in Western European countries such as Portugal, from which Brazil received the most migrants [50,51]. To a lesser extent, other Y chromosomes belonged to clade F-M89 (15.6%), from which other common European Hgs (such as I and J, that have been frequently described in other *quilombos*) derive [7].

The African paternal contribution accounted for 39.4% in Feixo and 25% in Restinga. Notably, no Y-chromosomal lineages from the African A or B clades were detected in either community, as all Y chromosomes analyzed exhibited derived states for at least one of the tested SNP markers.

Overall, 35.6% of the Y chromosomes belonged to clade E-P152 (Table 1). P152 defines the basal clade E (also defined by the SNPs M96 and M40) of African origin and from which most African haplogroups derive [42]. Of them, 17.8% (18.2% in Feixo and 16.7% in Restinga) were also positive for the derived allele of SNP M35, which defines Hg E1b1b1 (Table 1). Only three chromosomes (6.7% of the total) presented the derived state of M2, the mutation that defines Hg E1b1a1 (Table 1).

Only 6.7% of the chromosomes (all of them detected in Feixo) belonged to Hg Q-M242, a Pan-American Q lineage from which most Indigenous American lineages derive [52]. All Q-M242 chromosomes were also positive for Q1b-M346 and its derived lineage Q1b1a1a-M3. Q1b1a1a-M3 is an autochthonous Indigenous American Hg and the most frequently observed in Natives from South America [53].

### 3.6. Molecular Variance (AMOVA) and Diversity Indices of Maternal Lineages in Quilombo Populations

We ran a molecular variance analysis (AMOVA) based on the polymorphic sites studied in this work to evaluate the degree of differentiation of the two *quilombos*. Interestingly, we did not detect a significant molecular variance between populations in either the mtDNA (Monte-Carlo test of significance *p*-value = 0.49) or Y chromosome data (Monte-Carlo test of significance *p*-value = 0.99), as most variance is condensed between the haplogroups.

In addition, we assessed the genetic diversity parameters of the African maternal fraction from these two communities. We observed that, despite its minor African maternal contribution when compared to Restinga (Table 2), Feixo presented higher values for all the parameters evaluated. Compared to the urban centers of Paraná, the haplotype diversity indices in the *quilombos* were smaller (*H*: 0.997) [54].

### 3.7. Maternal but Not Paternal Lineages Reflect Self-Declared “Color”

To address the limited data on how self-declared “color” relates to ancestry in rural traditional communities of African descent, we assessed its association with uniparental ancestry in Feixo and Restinga.

In the two *quilombo* communities studied, the vast majority of participants self-identified as Negros—either Black (Preto) or Admixed (Pardo)—(93.1% in Feixo and 91.4% in Restinga). The distribution of categories differed between communities: in Feixo, most declared themselves Pardo (63.5%), while in Restinga the majority identified as Preto (57.4%) (Fisher’s exact test *p*-value = 0.0008185). A minority reported being White in both *quilombos* (7% in Feixo and 8.5% in Restinga). These proportions are also highly divergent from those reported in the last census for the general population of Southern Brazil where 78.2% people self-declared White, 20.9% Admixed, and 5.4% Black (Pearson’s Chi-squared *p*-value =< 2.2 × 10^−16^) [4].

**Figure 4:**
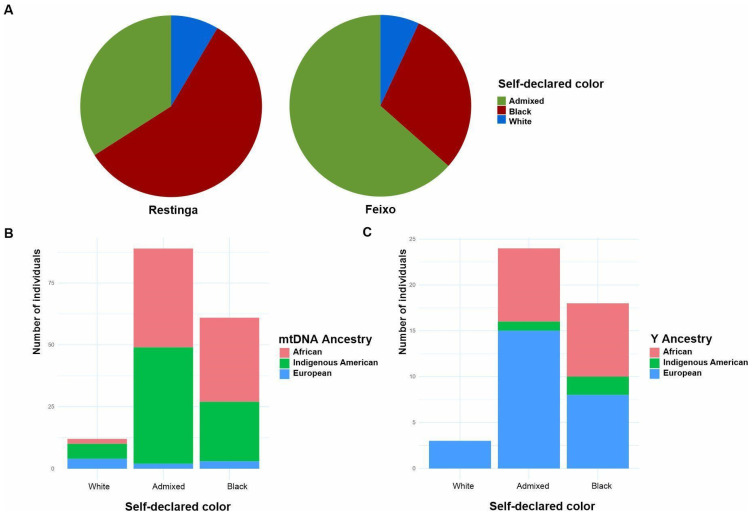
Self-declared color proportions in *quilombos* from Southern Brazil are associated with maternal but not paternal ancestry. (**A**) Self-declared color proportions in the studied *quilombos* (**B**) Mitochondrial ancestry proportions according to self-declared color (**C**) Y chromosome ancestry proportions according to self-declared “color”.

When the uniparental ancestry of the participants was compared to their self-declared color, it was associated with maternal ancestry in both communities (Fisher’s exact test *p*-value = 0.006899 in Feixo and *p*-value = 0.005867 in Restinga). Individuals who identified as Black exhibited predominantly African ancestry (55.7% African, 39.3% Indigenous American, and 4.9% European ancestry). In contrast, self-declared Whites showed a majority of Indigenous American maternal ancestry (50%), followed by European (33.3%) and African (16.7%). Similarly, those identified as Admixed also had predominantly Indigenous American ancestry (52.8%), though with a significant African component (44.9%) and minimal European ancestry (2.2%) (Figure 4).

On the other hand, paternal ancestry did not show a significant association with self-declared color in either community (Fisher’s exact test *p*-value = 0.47). Notably, European paternal ancestry predominated across most color categories—100% among individuals self-identified as Whites and 62.5% among those identified as Admixed. The proportion of European paternal ancestry was equivalent to the African ancestry only among individuals who self-identified as Blacks, with both accounting for 44.4%.

## 4. Discussion

When examining maternal ancestry patterns across Brazil, the results observed in Feixo and Restinga align well with national trends. The predominance of Indigenous American and/or African maternal lineages is a consistent feature in most Brazilian populations [6,41]. In particular, the substantial Indigenous American contribution found in Feixo mirrors findings from previous studies in urban populations of Paraná, where maternal Indigenous American ancestry has been reported to range from 38.4 to 49.2% [54,55].

Along the same line, Indigenous American maternal ancestry ranges from 10% to 60% in the Brazilian *quilombos* studied so far [6]. However, the elevated proportion observed in Feixo is only comparable with that observed in *quilombos* from the Amazon region [16,17,18]. In the Amazon, the high degree of Indigenous American admixture in *quilombos* correlates with that region concentrating the greatest density and diversity of Native American populations in Brazil (Brazilian Socioenvironmental Institute; https://pib.socioambiental.org (accessed on 23 July 2023). By comparison, the maternal ancestry of *quilombos* from southern Brazil remains largely unexplored [7]. Nonetheless, an average of 4.7% Indigenous American paternal ancestry (with most communities showing null Indigenous American paternal contribution) and an average of 19.3% autosomal Indigenous American ancestry observed in *quilombos* from southeastern Brazil (mainly from the Ribeira Valley, a region geographically close to Paraná), suggests a predominantly female-mediated Indigenous American gene flow in those populations [19,20].

### 4.1. Have Different Indigenous American Ethnic Groups Contributed to the Formation of Feixo and Restinga?

Among the original populations of the region where the *quilombos* are located—Guarani Mbya and Guarani Ñandeva (Tupian linguistic branch), Kaingang and Xocleng (Macro-Je branch)- genetic data are currently available only from Guarani and Kaingang ethnic groups [56,57]. The absence of Hg D in both *quilombos* is strongly consistent with the contribution of Kaingang women, as Hg D has never been detected in that ethnic group, whereas it has been found in most Guarani populations studied so far [57,58]. Furthermore, the highest Hg C prevalence in Feixo and the Kaingang also points in that direction [57]. On the other hand, the majority of Hg A in Restinga could suggest the contribution of Guarani women, as that Hg prevails in most Guarani populations studied so far [58]. In agreement with this hypothesis, oral narratives from Restinga recall the incorporation of a Guarani woman into the community. The frequency of Hg D varies greatly in Guarani populations (0–50%) as well as the frequency of B (0–17%), which was the least frequent Hg in both *quilombos* [58].

The role of Indigenous women in the formation of *quilombos* has been the subject of ongoing historical and anthropological debate in Brazil. On the one hand, the political articulation and cooperation of African descendants with Indigenous American Peoples, as well as with other minorities discriminated against in colonial society, have been repeatedly cited [2,5]. Other records include reports of the capture of Indigenous American women by *quilombolas* [2,5]. The *quilombo* Feixo, located in the same area, shows a high frequency of maternal Indigenous American ancestry, with haplogroup C notably predominant. This pattern coincides with the distribution observed in nearby urban centers, where haplogroup C is also frequent [54,55], suggesting that the genetic composition of Feixo may reflect both a strong Indigenous contribution and gene flow from neighboring populations, as previously reported for that community [9]. This interpretation is further supported by, and consistent with, the higher diversity indices observed in Feixo.

### 4.2. Differential African Genetic Inputs May Explain the Contrasting Ancestries of Feixo and Restinga

Although both communities are located in the same city, the differences in haplogroup composition between Feixo and Restinga suggest they may have received distinct African genetic inputs. Most haplogroups were exclusive to each community, with only one haplotype—belonging to haplogroup L2c2b1b—shared between them. Based on our phylogeographic analysis and the existing literature, we explore the plausible geographic sources of these lineages.

Haplogroups L1b and L1c—detected in Feixo (L1b) and Restinga (both L1b and L1c)—display different distribution patterns across Africa. Sub-branches of L1b are particularly diverse and prevalent in West Africa, whereas L1c lineages are found in both West and West-Central Africa [59]. L1c-derived branches are present in Central African hunter-gatherer and Bantu-speaking populations and are thought to have originated from a shared ancestral group [60]. The L1b1a10 and L1b1a+189 lineages identified in Feixo show close matches with both West African non-Bantu and Central African Bantu populations, supporting the idea of multiple potential sources for these haplogroups in the Feixo gene pool. Conversely, the exclusive presence of L1c1b and L1c1b1 in Restinga—matching haplotypes found among Bantu-speaking, Khoe-Kwadi-speaking, and hunter-gatherer groups from Angola and Zambia—is consistent with a possible southern African Bantu origin [45].

Haplogroup L2a1, which originated in West Africa, is among the most complex sub-branches of L2a [49], encompassing lineages found across both African and non-African regions. In Restinga, the high frequency and low diversity of the L2a1l lineage suggest a strong founder effect, likely introduced by a small number of maternal ancestors. While L2a1l is believed to have originated in West Africa, it is currently distributed across both West and Central Africa [49]. Because most sub-branches of L2a1l are defined by mutations outside the control region, it is difficult to pinpoint the exact origin of this lineage in Restinga [61]. However, our phylogeographic analysis points to both North and West Africa as plausible sources. In a similar pattern, L2a1c2a—also exclusive to Restinga—originated and is distributed in West and Central Africa [49]. Considering that North Africa played a minimal role in the transatlantic slave trade, and due to historical gene flow between North and West Africa, West Africa remains a plausible main source of the L2a1l and L2a1c2a lineages found in Restinga [35].

The lineage L2c2b1b—shared between Feixo and Restinga—is a derivative of the ancestral L2c clade, which originated in West Africa. However, L2c2b1b is specifically associated with Southern African populations and has been linked to the Bantu expansion [49]. Notably, this lineage has not been observed in Eastern Africa [49], which is consistent with our phylogeographic findings and is compatible with a Southern African origin for this haplotype in both *quilombos*.

Although the ancestral L0a clade originated in Eastern Africa, most of its derived lineages—such as L0a1 and L0a2—are currently more common in Central Africa [49]. This includes 75% of L0a-derived lineages. An exception is L0a2a2a, which may have an Eastern African origin but was likely assimilated by Bantu populations during their southward expansion [49]. As L0a2a2a is defined by a mutation (C13116T) outside the control region, which was not analyzed in this study, it remains possible that the L0a2a2 lineage found in Feixo may belong to this branch [61]. In either scenario, the haplotype detected in Feixo is consistent with an origin in Central or Southern African Bantu-speaking populations.

Haplogroup L3d3a—observed in Feixo—is part of the broader L3d clade, which occurs at low frequencies across African Bantu and Khoisan populations. It reaches its highest frequency in southwestern Africa, particularly Namibia [62]. There, sub-branches of L3d3a1 are notably common in Herero and Himba pastoralists (47% and 38%, respectively), as well as among their Khoisan-speaking neighbors, the Damara (63%) [62]. These lineages are thought to have originated among Khoe-Kwadi-speaking pastoralist groups and were later incorporated into Bantu populations through gene flow [62]. Since L3d3a sub-lineages are also defined by mutations outside the control region [61], we cannot classify the haplotype observed in Feixo. Nevertheless, given that Khoisan-speaking populations were unlikely to have been transported to Brazil during the transatlantic slave trade, a Central or Southern African Bantu origin for this lineage in Feixo represents a plausible explanation [63].

The Correspondence Analysis is consistent with our in-depth analysis of Hg composition. It is important to note that, as *quilombos* are populations typically formed by a small number of individuals, they are subject to strong genetic drift, particularly the founder effect [2]. A certain degree of isolation—especially during the first generations after the formation of a *quilombo*—could potentially increase endogamy in such populations [64]. Thus, the patterns observed are strongly influenced by the enrichment in specific lineages.

On the other hand, the clustering of the big urban centers could reflect a broader picture of the mitochondrial lineages introduced by the transatlantic slave trade in Central-Southern Brazil, as the three regions analyzed, according to historical records, received enslaved Africans mainly through the port of Rio de Janeiro. Conversely, the position of *quilombos* could have been more influenced by the effect of genetic drift in their formation, as is further reinforced by the lower diversity indices detected in both *quilombos* when compared to other urban centers from the region. However, it is essential to highlight that this approach is limited by the lack of data for most Brazilian *quilombos*—so far, genetic studies have included only around 93 of the 7666 extant African-derived communities, representing just about 1.6% of the total—and by the different genetic markers used across studies (coding SNPs, HVR1, or the complete control region) [4,7].

### 4.3. Paternal Lineages Reveal a Marked Sex Bias in the Formation of Feixo and Restinga

The paternal ancestry of Feixo and Restinga follows a pattern previously observed in other *quilombos* from Brazil [7]. In Brazil, as in other European colonized populations, the admixture process was largely biased by a predominantly male-mediated European gene flow, contrasting with Indigenous American and African contributions being frequently female-mediated [16,55]. The relative proportion of the last two ancestries usually varies more according to the formation of the population [7,55]. This sex bias has been largely associated with the countless reports of rape of Indigenous American and African women by European men and their descendants, as well as with the asymmetric relation of power between the sexes in the American colonies [65,66,67,68].

The same pattern has been frequently observed in other Brazilian *quilombos*, especially in Southeastern Brazil, as well as in other African-derived communities formed by ex-enslaved persons in America [7,20,69,70]. In particular, two scenarios could have determined the excess of European Y chromosomes in Feixo and Restinga. First, that could result from the sexual exploitation of African enslaved women, possibly used as reproducers of the slave labor force as has been previously reported [71]. In agreement with these historical accounts, oral narratives from Restinga also recall episodes of sexual violence against enslaved women. That hypothesis is reinforced by the historical records reporting that most enslaved African descendants in Paraná were born in Brazil [13]. Secondarily, it can be caused to some degree by more recent gene flow from surrounding urban regions [10].

### 4.4. The African Male Fraction Is Enriched in Eastern/Southern African Haplogroups

The majority of African Y-chromosome haplogroup diversity falls within clades A, B, and E. Clade A is considered the most ancient and prevails in eastern and southern Africa [42,72]. B lineages have a broader distribution over Sub-Saharan Africa but with the highest prevalence among Central African hunter-gatherers [63]. However, the greatest proportion of African Y-chromosome diversity is represented by sub-lineages of Hg E [73].

Haplogroup E1b1b1-M35, to which most E haplotypes in the *quilombos* belonged, has an eastern African origin and is one of the most broadly distributed E clades [74]. Its derived lineages reach high frequencies in a broad area from northern and eastern Africa to Europe and western Asia [74]. Even though specific E1b1b1-derived lineages (V6, M78, M81, M123, and M293) have been reported at high frequencies in European populations, the same lineages are also present in northern, eastern, and southern Africa [43,65,66]. Some of its terminal branches are restricted to Eastern African pastoralists and Cushitic populations from the Horn of Africa [74]. Furthermore, its paraphyletic branch, E1b1b1-M35*(xV6, M78, M81, M123, M293), is observed at low frequencies in Western Africa and it is common in eastern and southern Africa, both in Bantu, as in Nilo-Saharan, and Afroasiatic populations, but it is very rare in Europe [74,75,76].

It is important to highlight that all the participants carrying Hg E1b1b1-M35 reported that their fathers were also born in the *quilombo*. Thus, despite its phylogeographic ambiguity, we classified all E chromosomes, including the E1b1b1-M35 lineages, as African, based on the strong links of *quilombos* with that ancestry. E1b1a1-M2, representing a minor fraction of E haplotypes in the *quilombos*, and its derived lineages are the most common in Niger-Congo populations, such as Bantu-speaking, among other Sub-Saharan African groups, such as the non-Bantu Khoisan (Southern Africa) [77].

While most genetic studies in Brazil emphasize lineages from Western Africa, our analysis of these *quilombos* reveals a less common genetic profile [78]. The high frequency of Hg E1b1b1-M35 and E(xM35, M2) is compatible with a significant male-biased contribution from Eastern/Southern Africa, a pattern rarely highlighted in Brazilian population genetics, but consistent with broader genomic evidence [68]. Notably, these haplogroups are prevalent in groups such as the Hema (Nilotic speakers from northeastern Congo) and Southeastern Bantu populations [76,79], revealing understudied sources of the African diaspora in Brazil beyond the dominant Western African narrative.

### 4.5. What Self-Declared “Color” Reveals About Genetic Ancestry in Quilombo Communities

In Brazil, a large part of anti-racist social policies is based on self-declaration of color, officially referred to as “color or race” (raça e cor, in Portuguese), relying here on the social dimension of the latter concept [80]. Thus, government surveys and censuses use the phenotypic categories that the Brazilian Institute of Geography and Statistics (IBGE) defines. These categories are Preto, Pardo, Branco, Amarelo, and Indigena, translated here as Black, Admixed, White, Asian, and Indigenous. It has been demonstrated that a complex interplay between culture, genealogy, appearance, and shared history of slavery or discrimination plays a role in self-identification in Brazil [80,81,82].

Previous studies have evaluated the association of self-perceived ethno-racial categories with autosomal and uniparental ancestries in urban populations [81,82,83]. These studies have shown that self-declared perception of ancestry depends heavily on non-biological cultural factors which can vary according to the population studied [82]. To evaluate whether self-declared “color” reflects biological ancestry in *quilombo* communities—where this declaration holds social, political, and biomedical importance—we analyzed its association with uniparental genetic markers.

The significant divergence in self-declared “color” proportions between Feixo and Restinga reflects both their distinct genetic ancestries and Brazil’s sociopolitical context of racial identification. Restinga’s predominant Black (Preto) self-identification aligns with its high African maternal ancestry, while Feixo’s majority Pardo (Admixed) identification corresponds to its elevated Indigenous American maternal component—a pattern consistent with observations in urban Brazilian populations [83]. These proportions starkly contrast with the general Brazilian population’s distribution, underscoring how *quilombo’s* unique historical formation processes shaped their demographic trajectories. Beyond genetic correlations, this categorical divide engages with a fundamental tension in Brazil’s racial classification system. The Black Movement has long contested the IBGE’s separation of Preto and Pardo, arguing it fragments the population experiencing structural racism and weakens claims for reparative policies [26]. Our findings demonstrate that while these categories capture biological ancestry differences at the community level, their political implications remain inseparable from their demographic usage-highlighting how self-identification serves simultaneously as an ancestral descriptor and a potential act of collective positioning.

On the other hand, since European paternal ancestry predominates in most Brazilian populations investigated so far, mitochondrial lineages likely capture Brazilian population substructure more effectively [7,55,84,85]. This hypothesis may be particularly relevant for communities with heightened ancestral awareness due to socio-political factors, such as *quilombos*, where self-declared ethnic identity is required for legal recognition and land rights preservation [9].

## 5. Conclusions

Winds and shorter distances minimized the travel between the ports of West Central and Southeastern Africa (mainly Angola and Mozambique) and Rio de Janeiro in the Southeast of Brazil [35]. Rio de Janeiro is expected to be the main entrance port for the Africans brought to Paraná state. That is because it is thought that most Africans came to Paraná enslaved along the livestock-associated movements between the states of Rio Grande do Sul and São Paulo. Also, they could have been brought by the incoming migrants from São Paulo, representing a great fraction of the immigration during the early formation of the urban centers of Paraná [12]. Furthermore, some historical records narrate the entrance, by the port of Paranaguá (in Paraná state), of ships from Angola and Mozambique [43]. The last records represent events after the English prohibition of the enslavement of African people (1831), which caused the traffic of Africans to continue illegally through minor and less monitored ports from Brazil, such as the port of Paranaguá [43]. At that time, the traffic from Mozambique intensified [35].

Considering both historical and genetic data, our findings reveal a previously underappreciated complexity: within the same geographic region of Brazil, we identified patterns compatible with distinct African regional contributions across *quilombo* communities, a diversity often obscured by broad-scale analyses. While Feixo exhibited maternal lineages pointing to Southern/Central/Southeastern Africa (in line with regions historically associated with Angola, Congo, and Mozambique) and paternal E-M35 haplogroups compatible with Central-Eastern/Southern African contributions, Restinga showed mitochondrial lineages with affinities to West African populations. Genetic drift could have substantially modified the frequencies of certain lineages, increasing expressively the estimated proportion of Western haplotypes in both *quilombos*. This mosaic of African ancestries within a single Brazilian region highlights the limitations of homogeneous narratives of the transatlantic slave trade’s genetic legacy, even though no significant population-level differentiation was detected. These results demand finer-scale models that capture both regional diversity and microevolutionary dynamics in African-derived populations.

The proportion of mitochondrial Indigenous American Hgs detected suggests a great participation of Kaingang women in forming the *quilombos*, especially Feixo. Restinga showed a smaller Indigenous American maternal fraction, with haplogroup proportions suggesting a contribution from Guarani populations. However, the lack of genetic data from other Indigenous American ethnic groups that lived in the state of Paraná limited the identification of specific populations.

Both populations present smaller indices of genetic diversity when compared to the urban populations of Paraná, as expected of populations subjected to strong founder effects. Although these two *quilombos* did not present a significant molecular differentiation, the smaller diversity indices estimated for Restinga suggest a stronger effect of genetic drift in that population.

This study represents the first approach to the allelic architecture and demographic history of Afro-diasporic communities from the region of Paraná, in Southern Brazil, a region where the African roots have been largely hidden. We also provide the first exploratory estimates of the sub-continental African and Indigenous American contributions to Brazilian *quilombos* based on uniparental markers. Furthermore, it adds new clues to the big picture of the transatlantic traffic of human beings, digging deeper into the history of a previously unexplored Brazilian slave region.

Final considerations

Here we investigate for the first time the genetic ancestry of two *quilombos* from Paraná state and perform an in-depth analysis of their uniparental African contributions. In addition, we conducted the first thorough examination of maternal Indigenous American lineages in a Brazilian *quilombo*, integrating genetic and historical data to explore interactions with indigenous groups.

It is important to emphasize that these results do not provide definitive evidence regarding the origins of the founders of the two *quilombos* studied, nor do they indicate significant population-level differentiation between the two *quilombos*. Both mtDNA and Y chromosome lineages are distributed across broad regions of Africa due to various migration events, making it only possible to analyze the patterns that emerge from the observed frequency differences [86,87,88,89,90,91,92,93,94,95,96,97]. Nonetheless, we offer informed hypotheses about plausible sources of the lineages found in Feixo and Restinga, integrating genetic data with existing historical records to contribute to the understanding of the history of these communities.

## Figures and Tables

**Table 1:** Frequency of mtDNA and Y chromosome haplogroups detected in the two studied *quilombos*.

Haplogroup	*Quilombos*		Feixo		Restinga	
mtDNA	n	%	n	%	n	%
A	16	9.9	12	10.4	4	8.5
B	6	3.7	5	4.3	1	2.1
C	55	34.0	53	46.1	2	4.3
D	0	0	0	0	0	0
Total Indigenous American	77	47.5	70	60.9	7	14.9
M	1	0.6	1	0.9	0	0
N	8	4.9	5	4.3	3	6.4
Total European	9	5.6	6	5.2	3	6.4
L	76	46.9	39	33.9	37	78.7
L0a2a2	1	0.6	1	0.9	0	0
L1b1a+189	4	2.5	4	3.5	0	0
L1b1a10	4	2.5	3	2.6	1	2.1
L1c1b	1	0.6	0	0	1	2.1
L1c1b1	1	0.6	0	0	1	2.1
L2a1+143+16189+(16192)	2	1.2	2	1.7	0	0
L2a1+16189+(16192)	18	11.1	18	15.7	0	0
L2a1c2a	1	0.6	0	0	1	2.1
L2a1l	32	19.8	0	0	32	68.1
L2c2b1b	7	4.3	6	5.2	1	2.1
L3d3a	5	3.1	5	4.3	0	0
Total African	76	46.9	39	33.9	37	78.7
Y chromosome						
Q	3	6.7	3	9.1	0	0
Q-M3	3	6.7	3	9.1	0	0
Total Indigenous American	3	6.7	3	9.1	0	0
R	19	42.2	13	39.4	6	50.0
F	7	15.6	4	12.1	3	25.0
Total European	26	57.8	17	51.5	9	75.0
E	16	35.6	13	39.4	3	25.0
E-M35	8	17.8	6	18.2	2	16.7
E-M2	3	6.7	2	6.1	1	8.3
Total African	16	35.6	13	39.4	3	25.0

**Table 2:** Population diversity indices for the two studied *quilombos*. *H*: Nei’s haplotype diversity index. π: Nucleotide diversity index. Hg: haplogroups. Hap: haplotypes.

Population	N	NHg	Nhap	Nhap/N	*H*	Variance	π	Variance
*Quilombos*	76	16	16	0.211	-	-	-	-
Feixo	39	7	10	0.256	0.812	0.002	0.131	0.005
Restinga	37	6	6	0.162	0.390	0.010	0.049	0.001

## Data Availability

The raw data supporting the conclusions of this article will be made available by the authors on request.

## Supplementary Materials

The following supporting information can be downloaded at: https://www.mdpi.com/article/10.3390/genes16121510/s1, Figure S1: Haplotype network of sub-branch L1b1a and derived lineages from Feixo and African reference populations; Figure S2: Haplotype network of haplogroup L1c1b and derived lineages from Restinga and African reference populations; Figure S3: Haplotype network of sub-branch L2a1 and derived lineages from Feixo and African reference populations; Figure S4: Haplotype network of sub-branch L2a1l and derived lineages from Feixo and African reference populations; Figure S5: Haplotype network of haplogroup L2a1c2a from Restinga and African reference populations; Figure S6: Haplotype network of L2c2b1b haplotypes from Feixo, Restinga, and African reference populations; Figure S7: Haplotype network of L3d3a and derived lineages from Feixo and African reference populations; Figure S8: Correspondence analysis biplot showing the distribution of the two *quilombos* studied and African reference populations according to the frequency of L-derived haplogroups; Figure S9: Correspondence analysis biplot showing the distribution of the *quilombos* Feixo and Restinga and other Brazilian *quilombos* and urban populations according to the frequency of L-derived haplogroups; Table S1: Primers used for the genotyping of sub-lineages of Y chromosome haplogroup E; Table S2: Diagnostic SNPs and haplotypes for identified Maternal Haplogroups; Table S3: Diagnostic SNPs for identified Paternal Haplogroups; Table S4: GenBank codes of sequences from reference African populations used in haplotype comparisons [36,46,49,60,62,87,88,89,90,91,92,93,94,95,96]; Table S5: Mitochondrial haplogroup frequencies in ancestral African reference populations used for correspondence analysis [97]. File S1: Supplementary Methods—General description of the bioinformatic workflow, including the functions and analytical steps used for haplotype inference, network construction (pegas), and Correspondence Analysis (FactoMineR/factoextra), with references to software manuals and parameter specifications.

## Abbreviations

The following abbreviations are used in this manuscript:

CA	Correspondence Analysis
Hg	Haplogroup
HVR	Mitochondrial hypervariable region
InDel	Insertion Deletion
MtDNA	Mitochondrial DNA
rCRS	Revised Cambridge Reference Sequence
SNP	Single Nucleotide Polymorphism
