# Supplementary material for: Tracing the Uncharted African Diaspora in Southern Brazil: The Genetic Legacies of Resistance in Two Quilombos from Paraná
Source: Genes (Basel). 2025 Dec 16;16(12):1510. doi: 10.3390/genes16121510 (PMC12733129; doi:10.3390/genes16121510)
Supplement: Supplementary file 1 [file genes-16-01510-s001.zip › File S1.pdf]

Supplementary Material: General Description of Code and Methods

1. Haplotype Network Construction using *pegas*

(References: pegas documentation: https://emmanuelparadis.github.io/pegas.html; CRAN manual, pp. 24–25, 42, 45)

We conducted the haplotype-based analyses using the R package pegas, following a standard workflow for sequence processing, haplotype inference, and network reconstruction.

Sequence formatting and DNAbin conversion

Aligned DNA sequences were imported and converted into DNAbin objects, the core sequence format used by the ape package for molecular and phylogenetic analyses.

DNAbin provides an efficient internal representation of nucleotide data, allowing fast computation of genetic distances and downstream population analyses (Manual pegas: pp. 24–25).

Haplotype inference

Haplotypes were identified using the haplotype() function in pegas, which collapses identical DNA sequences into unique haplotypes. This step produced a set of haplotypes characterized by their distinct polymorphism profiles, alongside absolute haplotype counts and relative frequencies. These frequency distributions were later used for population-level comparisons and structure analyses, "Haplotype extraction and frequencies", (Manual pegas: pp. 30).

Pairwise genetic distance computation

Genetic distances among haplotypes were estimated using the dist.dna() function from the ape package (Manual pegas, "Median-Joining Network", p. 42).

Distances were computed under the "N" model, which counts the number of nucleotide differences between sequences.

<u>Definition</u>:

dist.dna() computes a matrix of pairwise genetic distances using various evolutionary models (e.g., Jukes–Cantor, Kimura). It accepts DNAbin objects and includes options for variance estimation and pairwise deletion of missing data.

Haplotype network reconstruction

A reduced median spanning tree (RMST) was generated using the rmst() function (Manual *pegas*, "Minimum Spanning Tree and Network", p. 45).

This method identifies the shortest mutational paths among haplotypes, providing a graphical representation of mutational relationships and lineage connectivity.

Population-level haplotype frequency estimation

When population metadata was available, haplotype frequencies per population or region were computed using haploFreq() (Manual *pegas*, "Haplotype Frequencies With a Covariate", p. 25).

The resulting haplotype-by-population frequency matrix was used to contextualize the distribution of haplotypes across demographic groups.

Network visualization

Networks were visualized using the default *pegas* plotting system, where:

Node sizes were proportional to haplotype frequencies, and pie charts were used to display the contribution of each population to a given haplotype.

Graphical adjustments (e.g., label size, legend placement) followed recommendations from the *pegas* plotting guide:

(PlotHaploNet manual: https://emmanuelparadis.github.io/pegas/PlotHaploNet.pdf).

All analyses were run using typical parameters of pegas, with default distance options (model="N") and default plotting options, adjusting only label size and legend placement for clarity.

2. Correspondence Analysis using FactoMineR and factoextra

Correspondence Analysis (CA) was performed using the R package FactoMineR, and visualizations were produced with factoextra.

(References: FactorMineR documentation: https://cran.r-project.org/web/packages/FactoMineR/index.html; CRAN manual, pp. 6-7)

(References: factoextra documentation: https://cran.r-project.org/web/packages/factoextra/readme/README.html; CRAN manual, pp. 19-24)

The analysis consisted of:
• Input table preparation: A contingency table (populations x haplogroups) was used. Rows and columns with zero counts were removed prior to analysis.
• Correspondence Analysis (CA) computation:

- The CA was run using `CA()` with `ncp = 5` (number of dimensions retained) and `graph = FALSE`. Manual FactoMineR on page 6.
- Output of CA: Eigenvalues, row/column coordinates, contributions, and squared cosine values (cos^2) values. Manual FactoMineR on page 7.

• Visualizations: Biplots, row-only plots, and scree plots were generated using `fviz_ca_biplot()`, `fviz_ca_row()`, and `fviz_screeplot()`. When metadata was available, rows were colored by population or region.

Standard CA parameters from FactoMineR were used, with no data transformation beyond removal of empty rows/columns. Visualizations relied on default scaling and symmetrical representation of rows and columns.
