# Supplementary material for: Tracing the Uncharted African Diaspora in Southern Brazil: The Genetic Legacies of Resistance in Two Quilombos from Paraná
Source: Genes (Basel). 2025 Dec 16;16(12):1510. doi: 10.3390/genes16121510 (PMC12733129; doi:10.3390/genes16121510)
Supplement: Supplementary file 1 [file genes-16-01510-s001.zip › Genes_Supplementary figures_Tracing the African Diaspora in quilombos from Paraná_reviewed.pdf]

Supplementary Figures of the article: Tracing the Uncharted African Diaspora in Southern Brazil: the genetic legacies of resistance in two *quilombos* from Paraná

Authors: Iriel A. Joerin Luque[1,2], Isadora Baldon Blaczyk[2], Priscila Ianzen dos Santos[2,3], Ana Cecília Guimarães Alves[1,2], Natalie Mary Sukow[1,2], Ana Carolina Malanczyn de Oliveira[2],Thomas Farias de Cristo[2], Angela Rodrigues do Amaral Bispo[2], Aymee Fernanda Gros[2], Maria Letícia Santos Saatkamp[2], Victor Dobis Barros[2], Joana Gehlen Tessaro[2], Maria Eduarda da Silveira Costa[2], Luana Leonardo Garcia[2], Isabela Dall Oglio Bucco[2], Denise Raquel de Moura Bones[1,2], Sarah Elisabeth Cupertino[2], Letícia Boslooper Gonçalves[1,2,4], Alaerte Leandro Martins[5], Gilberto da Silva Guizelin[6], Adriana Inês de Paula[7], Claudemira Vieira Gusmão Lopes[8], Marcia Holsbach Beltrame[1,†*]

[1] Programa de Pós-graduação em Genética, Departamento de Genética, Universidade Federal do Paraná (UFPR), Curitiba, Paraná, 81531-990, Brasil.

[2] Laboratório de Genética Molecular Humana, Departamento de Genética, Universidade Federal do Paraná (UFPR), Curitiba, Paraná, 81531-990, Brasil.

[3] Programa de Pós-graduação em Medicina Interna, Hospital de Clínicas (HC), Universidade Federal do Paraná (UFPR), Curitiba, Paraná, 81531-990, Brasil.

[4] Laboratório de Imunogenética e Histocompatibilidade, Departamento de Genética, Universidade Federal do Paraná (UFPR), Curitiba, Brasil.

[5] Rede Mulheres Negras do Paraná (RMN-PR), Curitiba, Paraná, 80310-130, Brasil

[6] Programa de Pós-graduação em História, Universidade Federal do Paraná (UFPR), Curitiba, Paraná, 81531-990, Brasil.

[7] Departamento de Educação Física, Universidade Federal do Paraná (UFPR), Curitiba, Paraná, 81531-990, Brasil.

[8] Setor Litoral, Universidade Federal do Paraná (UFPR), Matinhos, Paraná, Brasil.

* Correspondence: marcia.beltrame@ufrgs.br

† Current affiliation: Departamento de Genética e Programa de Pós-graduação em Genética e Biologia Molecular, Universidade Federal do Rio Grande do Sul (UFRGS), Porto Alegre, Rio Grande do Sul, 91501-970, Brasil.

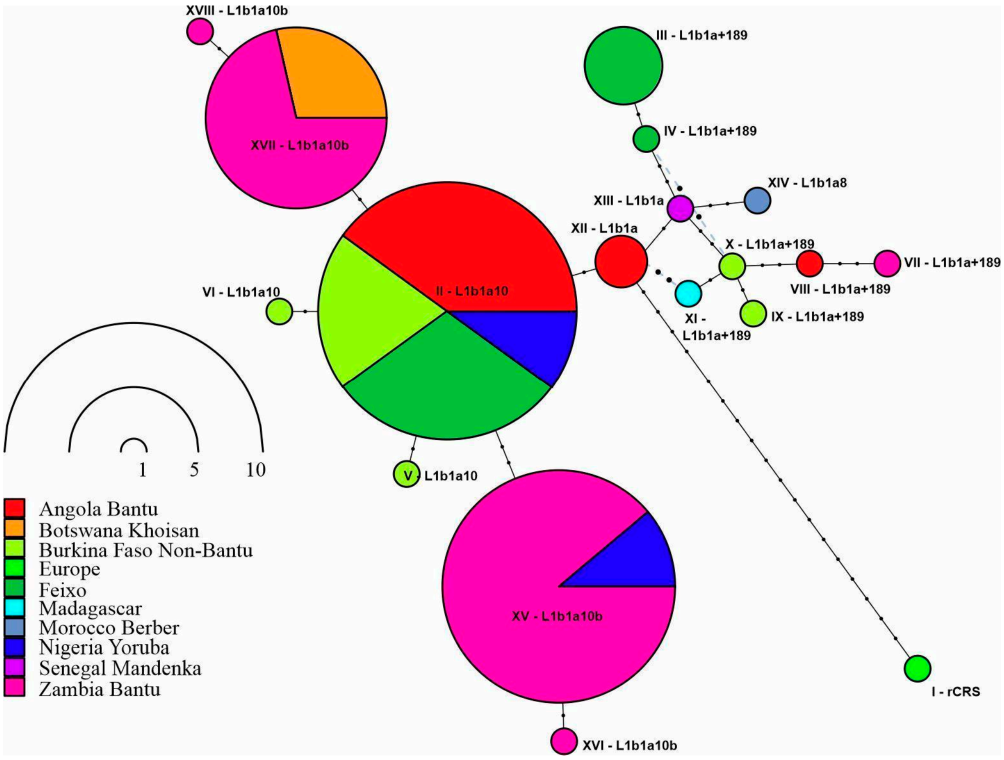

Figure S1 Haplotype network of sub-branch L1b1a and derived lineages from Feixo and African reference populations. Black circles on the network links represent mutational steps between the haplotypes.

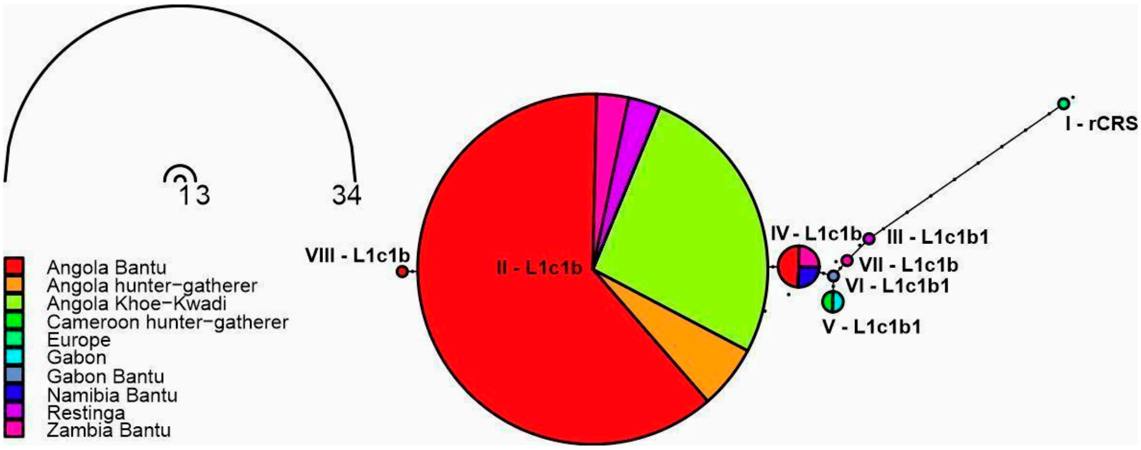

Figure S2 Haplotype network of haplogroup L1c1b and derived lineages from Restinga and African reference populations. Black circles on the network links represent mutational steps between the haplotypes.

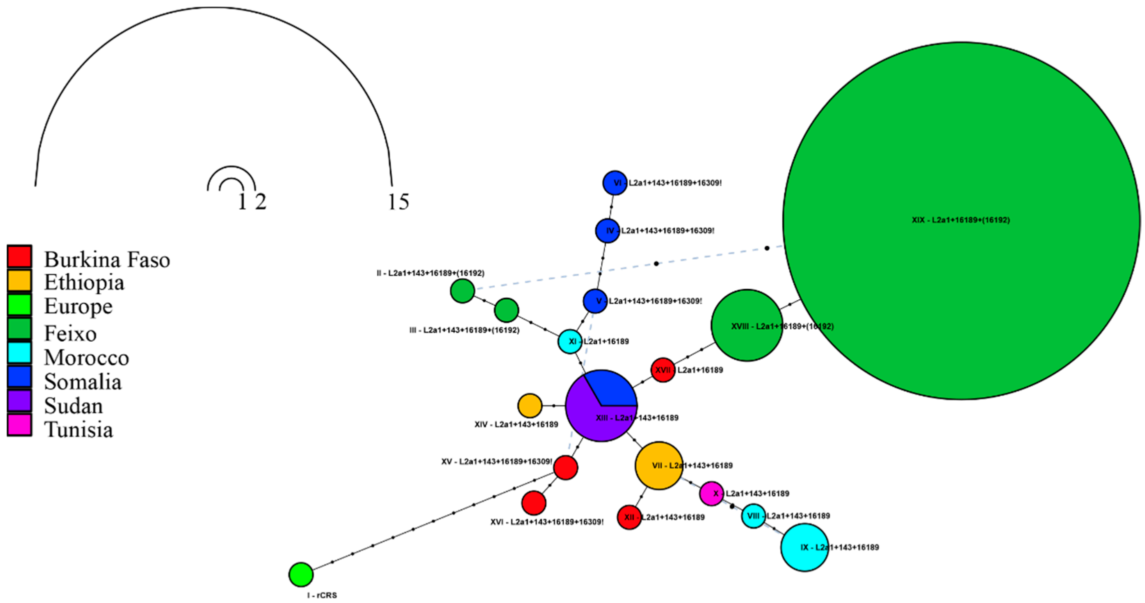

Figure S3 Haplotype network of sub-branch L2a1 and derived lineages from Feixo and African reference populations. Black circles on the network links represent mutational steps between the haplotypes.

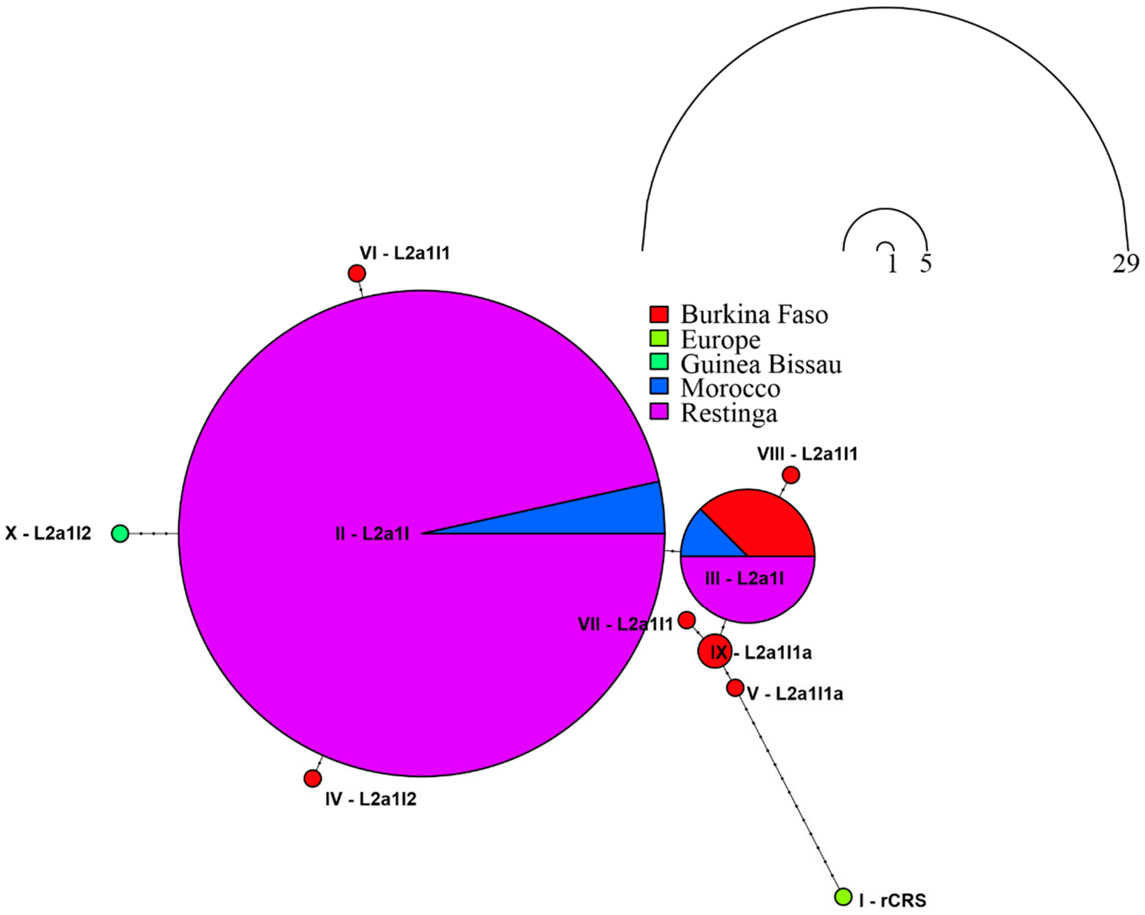

Figure S4 Haplotype network of sub-branch L2a1l and derived lineages from Feixo and African reference populations. Black circles on the network links represent mutational steps between the haplotypes.

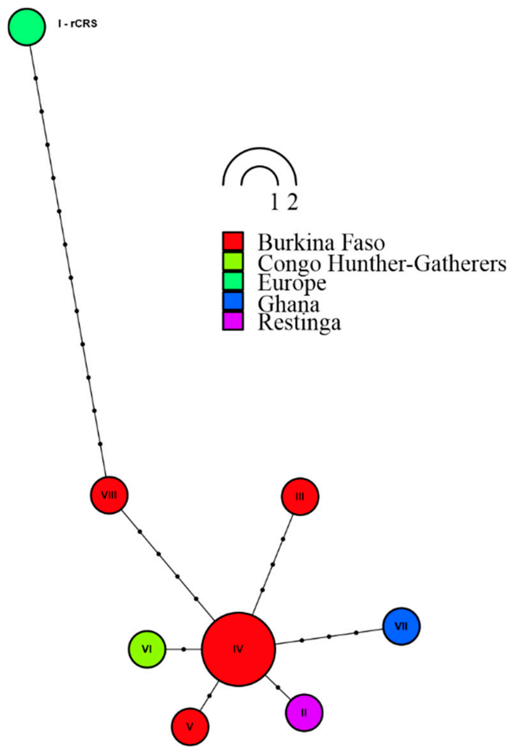

Figure S5 Haplotype network of haplogroup L2a1c2a from Restinga and African reference populations. Black circles on the network links represent mutational steps between the haplotypes.

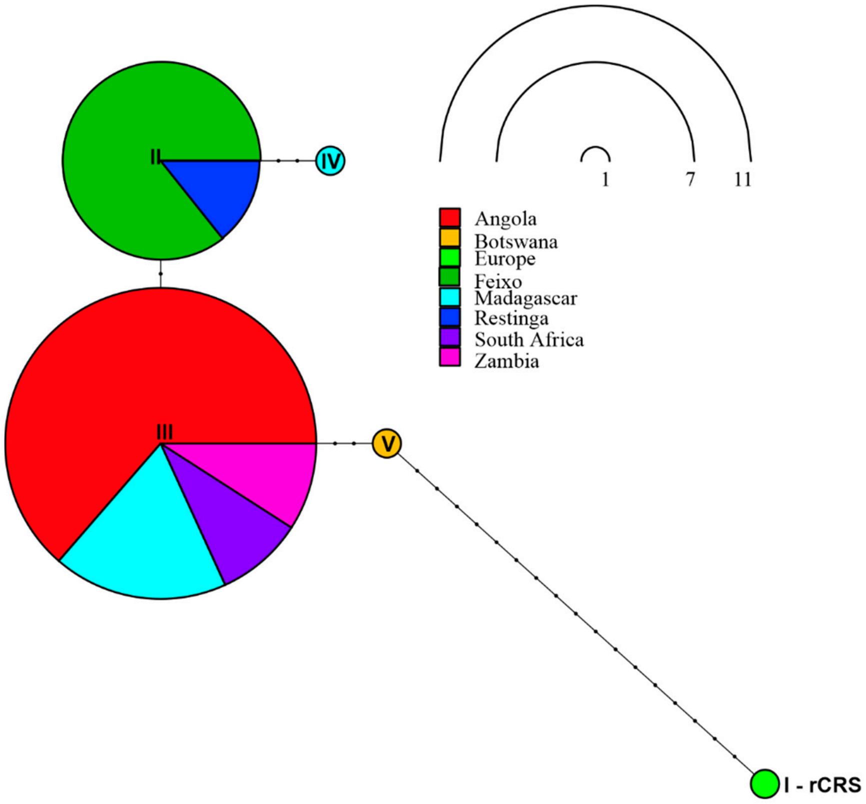

Figure S6 Haplotype network of L2c2b1b haplotypes from Feixo, Restinga, and African reference populations. Black circles on the network links represent mutational steps between the haplotypes.

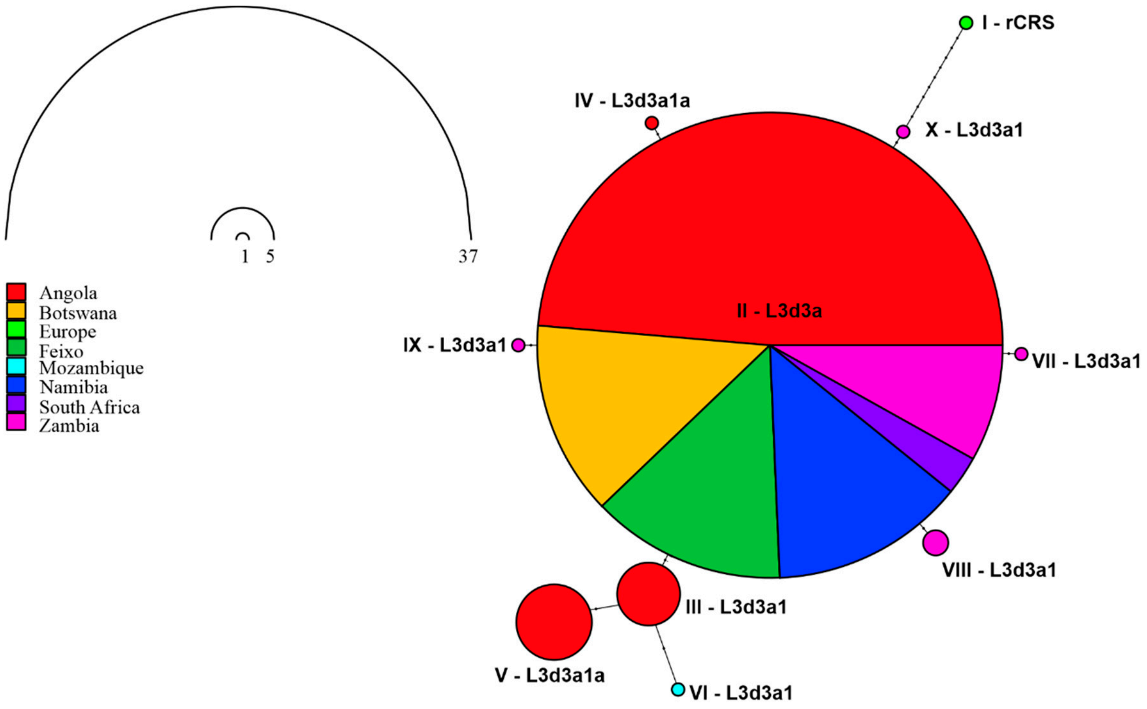

Figure S7 Haplotype network of L3d3a and derived lineages from Feixo and African reference populations. Black circles on the network links represent mutational steps between the haplotypes.

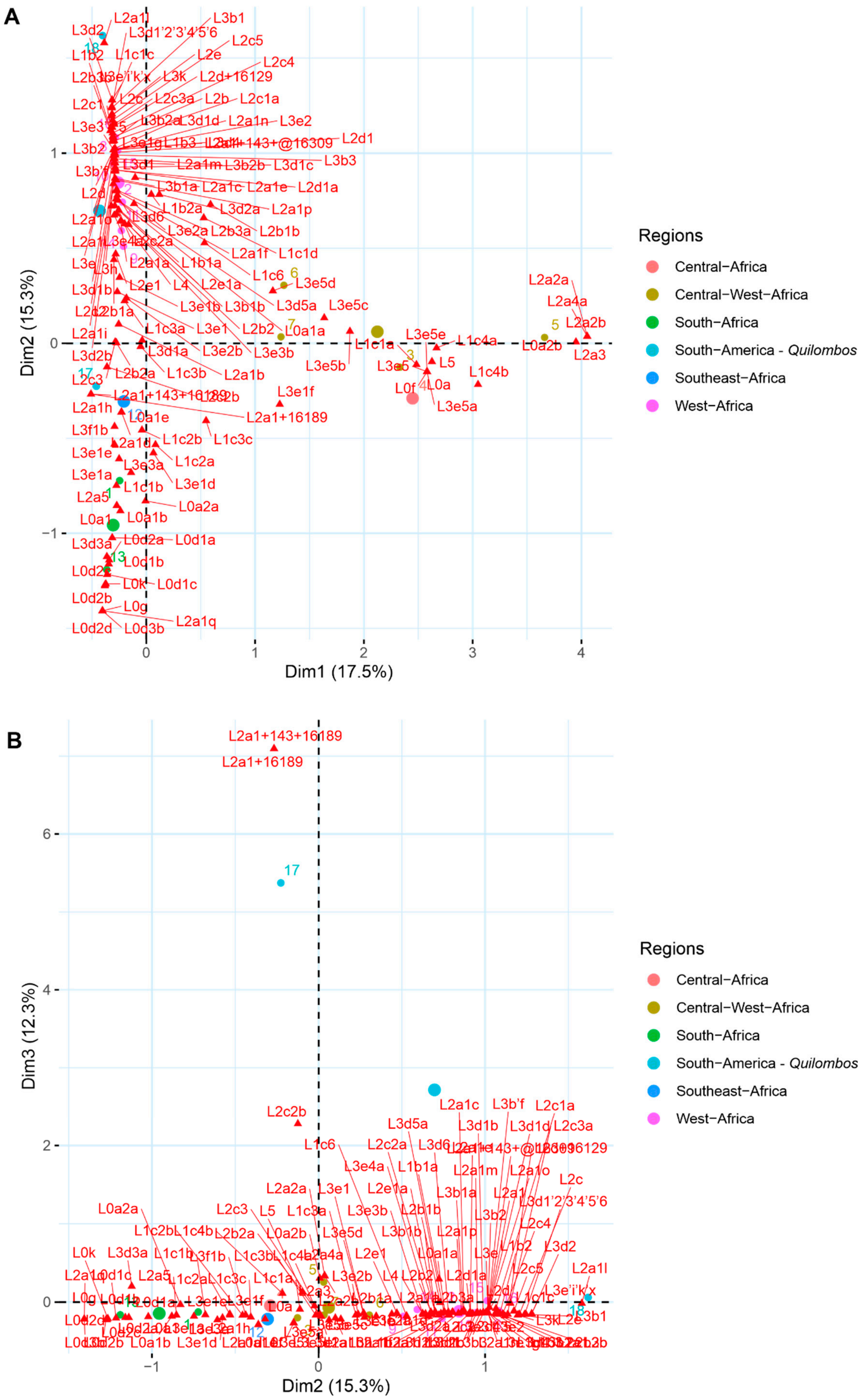
A
Dim1 (17.5%)
Dim2 (15.3%)
0
1
2
3
4
-1
Regions
Central−Africa
Central−West−Africa
South−Africa
South−America - Quilombos
Southeast−Africa
West−Africa
B
Dim2 (15.3%)
Dim3 (12.3%)
−1
0
1
2
4
6
Regions
Central−Africa
Central−West−Africa
South−Africa
South−America - Quilombos
Southeast−Africa
West−Africa

Figure S8 Correspondence analysis biplot showing the distribution of the two *quilombos* studied and African reference populations according to the frequency of L-derived haplogroups. A) Dimensions 1-2. **B)** Dimensions 2-3.

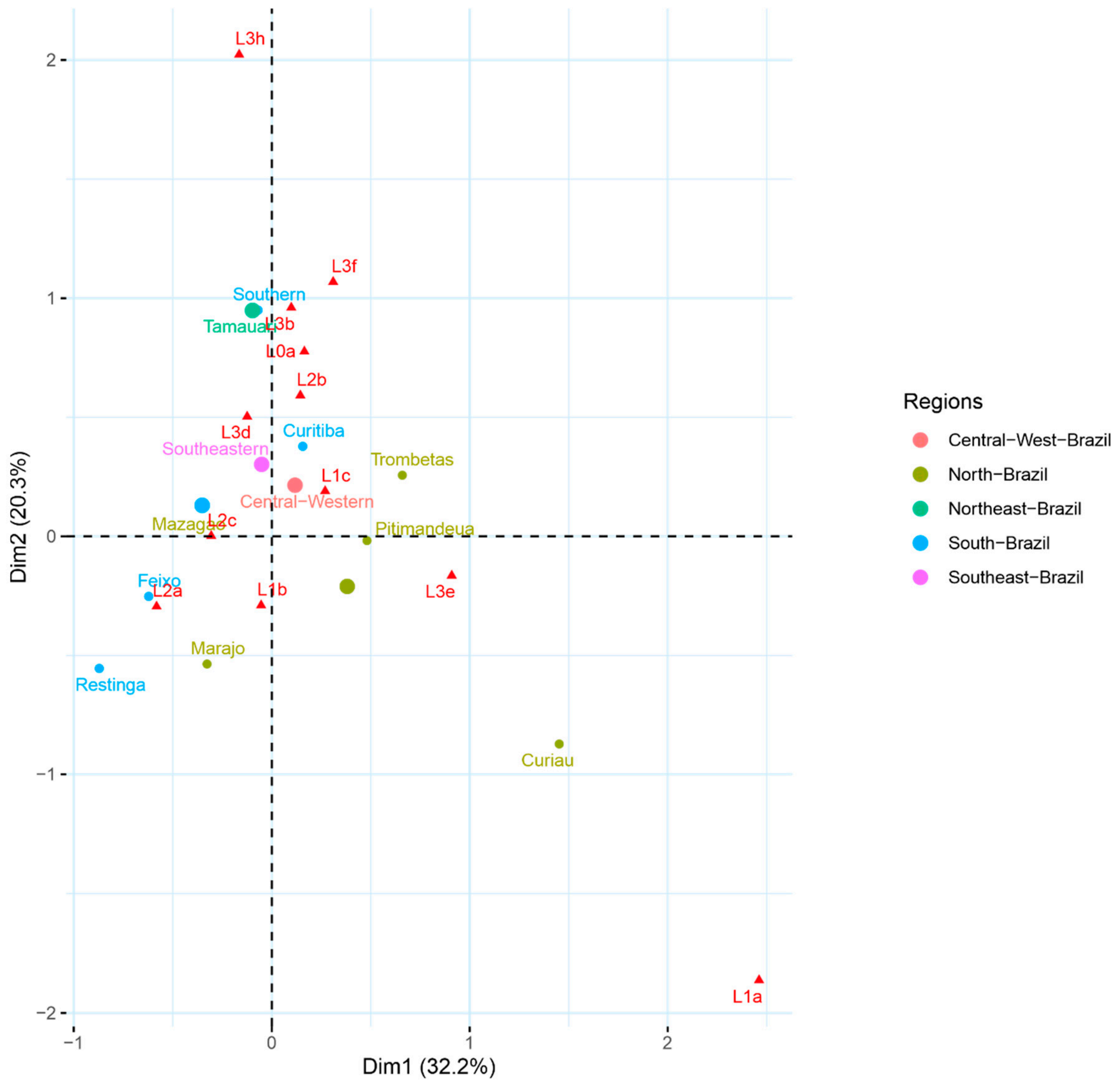

Figure S9 Correspondence analysis biplot showing the distribution of the *quilombos* Feixo and Restinga and other Brazilian *quilombos* and urban populations according to the frequency of L-derived haplogroups.
